# Exploring the Influential Factors of Personal Media Bloggers on Followers’ Continuous Following Intention Based on Relationship Marketing Theory

**DOI:** 10.3390/bs13050416

**Published:** 2023-05-16

**Authors:** Wenjie Qian, Jianhua Mao

**Affiliations:** 1 Faculty of Business and Management, Hong Kong Baptist University, Zhuhai 519087, China; 2 School of Liberal Arts and Law, Henan Polytechnic University, Jiaozuo 454000, China

**Keywords:** personal media, continued usage intention, relationship marketing, word of mouth, social presence

## Abstract

The use of personal media has become increasingly popular in recent years. However, gaining and retaining followers has become increasingly challenging, given the fierce competition among bloggers and the constant changes in personal media. In this context, this study aims to explore the factors that influence followers’ continued usage intentions toward personal media bloggers and strategies to improve their loyalty. Drawing upon the theory of relationship marketing, a structural model is constructed to examine the impacts and mechanisms of personal media bloggers’ attributes and communication on social presence, fanship, intention to use, and word of mouth. This research focuses on two dimensions of personal media bloggers’ attributes: expertise and attractiveness. A sample of 155 highly active personal media users in China was collected through a questionnaire for analysis and validation. The findings reveal that expertise and communication have positive impacts on followers’ intentions to continue following a blogger, while attractiveness has a significant, positive, and direct impact on word of mouth. Furthermore, this study shows that social presence and fanship play mediating roles in the effects of expertise and communication on followers’ usage intentions and word of mouth. The research results provide valuable insights for personal media operators and marketers seeking to improve followers’ loyalty and encourage potential users to become more loyal fans.

## 1. Introduction

The rapid development of personal media has rendered it the main channel of communication for internet users. According to the “50th Statistical Report on Internet Development in China” released by the China Internet Network Information Center (CNNIC), as of 2021, there were a total of 9.7 million people engaged in the personal media industry in China, and this number is still growing continuously [1]. With the emergence of personal media platforms such as Weibo, WeChat, TikTok, and Xiaohongshu, personal media in China has experienced exponential growth, and people’s consumption habits have shifted towards the internet and mobile terminals. The influence of personal media on user groups is constantly rising. Some users actively publish their own creative content on personal media platforms and have a certain number of followers. These users, with a large fan base, spread information or viewpoints that have a huge impact on their followers, and they are regarded as important sources of information by their followers. These people are called personal media bloggers [2].

Compared with traditional celebrity endorsements, personal media bloggers are considered to have more advantages in product placement and recommendation [3]. Therefore, with the rise of digital marketing, more and more companies have realized the commercial value of personal media bloggers and established cooperative relationships with bloggers to promote products or services as an important marketing channel [4]. This has given rise to the commercial model of personal media blogger marketing [5]. In this process, a large amount of capital has been invested into personal media, and personal media bloggers with a large number of fans, such as celebrities, can earn millions of dollars. In addition, these famous personal media bloggers not only profit from the content itself, but also from fans’ donations and sponsorship, making the relationship between bloggers and fans even more intimate. This has made the role and influence of bloggers increasingly complex and unique, rendering them a distinctive research group [6].

In the field of personal media marketing, previous research has focused on exploring the impact of blogger attributes on specific behaviors, such as consumer purchase motivation or purchasing behavior [5,7,8], or the limited exploration of the factors that make bloggers popular on specific platforms or in specific areas [9,10]. For example, bloggers increase the credibility of their content by utilizing their professional knowledge, gaining greater influence among their followers, and earning their trust [11]. Although the literature mentioned above has important promotional significance in expanding behavioral theories in the research on personal media blogger marketing, these studies focus more on marketing results and less on discussions that fully consider the advantages of blogger traits.

Moreover, the rapid development of personal media blogger marketing has also brought problems to the relationships between bloggers and their followers. For instance, some followers develop negative emotions toward the marketing information released by bloggers, and their emotions towards bloggers transition from intimacy to betrayal [12], leading them to unfollow the bloggers [13,14]. Due to the highly interactive nature of personal media, fans can respond to blogger content in real time through comments, chats, and other means, expressing their emotions and attitudes [15]. Therefore, negative content will further affect a blogger’s creativity and profits. As these behaviors do not incur significant alternative costs for followers [16], once the relationships between bloggers and fans are disrupted, the subsequent impact will be difficult to estimate.

From an industry perspective, personal media has now entered the era of capitalization, with frequent examples of large investments. Currently, tens of thousands of personal media users are undergoing industry consolidation, and those that lack core competitiveness, have low user retention, and cannot provide sustained high-quality content are likely to disappear. Those who have access to fan resources will begin to move towards capitalization and corporatization. This model exacerbates the pursuit of long-term stable traffic, more loyal fans, and competition in the field of personal media. Thus, the focus of competition among personal media bloggers is not only to attract more followers to increase their commercial influence but, more importantly, to establish good long-term relationships with their followers and enhance their loyalty. Specifically, it is necessary to understand the factors and mechanisms that influence the sustained use intentions of personal media platform users, which is of great significance for personal media managers to understand user needs and improve user retention. However, previous empirical studies have overlooked this important aspect [17].

In order to fill this gap, this article attempts to explore how to promote good relationships between bloggers and followers in order to enhance followers’ willingness to continue to follow bloggers, thus facilitating the development of personal media marketing activities. Ohanian’s information source theory emphasizes the beneficial effect of a celebrity’s professional knowledge and image charm on persuading followers [18]. Based on this, we consider expertise and attractiveness to be the core factors of a personal media blogger’s characteristics. In addition, personal media bloggers can interact with their followers through active likes and comments [19], and this level of interaction is higher than that of traditional celebrity endorsements [20]. Since this limited form of communication can help bloggers to establish closer relationships with their followers, we include bloggers’ communication behaviors in this study. Based on this, we propose two specific research questions: (i) Are a blogger’s expertise, attractiveness, and communication behaviors the factors that affect followers’ continued attention toward the blogger? (ii) What is the process mechanism by which these factors affect followers’ sustained attention? The power of word of mouth often brings a chain reaction and a significant increase in profits. Relationship marketing has moved away from a marketing concept that focuses on short-term sales performance goals. Instead, it focuses on building and maintaining long-term relationships with customers. Social media influencers provide users with diverse experiences, such as comments, private messages, and real-time chats, to establish and strengthen relationships with users. In this case, we believe that relationship marketing is an appropriate theoretical basis with which to address our research issues because it is important for explaining why certain characteristics of sellers are crucial in establishing good relationships with consumers, particularly in terms of their impacts on word of mouth and continued usage intentions [21]. Although relationship marketing is important, it has not been fully researched in the field of personal media. In addition, we introduce the concepts of fanship and social presence and verify their mediating effects. Fanship has an impact on people’s willingness to use media, their leisure activity arrangements, their relationships and acquaintances with family and friends, and even their own emotions [22], which is similar to the current real-life influence of personal media on followers. Social presence is an important psychological mechanism that explains why users are actively engaged in socializing and disseminating information on personal media platforms [23].

Our study contributes to the literature and research on enhancing the continued following of personal media followers. The theoretical contribution of this article is to expand relationship marketing theory to the personal media environment, exploring the most important factors allowing bloggers to enhance their fan loyalty and attempting to reveal the influence mechanism of blogger characteristics on sustained usage intention. This study makes a meaningful contribution to the existing literature on personal media marketing and relationship marketing. The practical contribution is that when shaping and utilizing their influence, bloggers themselves may need to pay more attention to long-term effects, focusing on cultivating and maintaining long-term relationships with their followers. In addition, when companies seek suitable blogger collaborations, they need to observe the characteristics of their potential collaborators and evaluate the relationship potential between the collaborator and their fans in order to optimize their marketing strategies.

## 2. Theoretical Background

### 2.1. Personal Media Blogger

The term “personal blogger” refers to users who share their own creative content through the internet to motivate their followers. These followers form a huge network of fans who are deeply influenced by the blogger’s content and opinions [24]. Due to the powerful influence and great commercial value of personal media bloggers, they have attracted extensive attention from the academic community. In recent years, research on personal media bloggers has been flourishing. Based on the comprehensive consideration of the quality and citations of the literature, we selected key studies on personal media bloggers in the past five years, as shown in Table 1. As we can see from the summary of the literature in the table, some studies have explored the influence of bloggers’ content and form on consumers’ purchase intentions from the perspective of content strategy [25], while others have classified personal media bloggers, compared bloggers with different numbers of fans, and studied the impact of sponsorship content disclosure on consumers’ purchase behavior [26]. There are also studies that have examined the performance of different types of personal media bloggers in terms of endorsement effectiveness [27], as well as studies that have focused on the marketing barriers of personal media bloggers and proposed solutions from the perspective of narrative strategy [28]. The latest research has examined consumer attitudes and purchase behavior from the perspective of the characteristics of bloggers’ credibility [29]. It can be seen that most studies on personal media bloggers focus on exploring factors that contribute to marketing outcomes, providing valuable insights to promote the development of personal media blogger marketing.

While these studies have provided some inspiration, we believe that the previous research has limitations. Firstly, when a blogger wishes to use their influence, especially by mobilizing their followers to generate economic benefits, this requires a foundation of good relationships between both parties to be established. Therefore, the relationship with their followers can be considered the cornerstone of a blogger’s long-term development. If a blogger neglects the management of their relationship with their fans, or even causes a rupture in trust between themselves and their followers, this undoubtedly will have a negative impact on the blogger [21]. Such negative impacts can greatly damage the reputation of the brand. To avoid such situations, bloggers need to establish good relationships with their fans, so as to reduce the risk of negative behaviors. However, the previous studies on personal media marketing have paid little attention to relationships and lack a discussion on how to fully utilize the advantages of relationships to bring better marketing results.

Secondly, while personal media blogger marketing is very popular on platforms such as Little Red Book and Weibo, with the rapid rise of personal media platforms such as TikTok and Bilibili, many brand owners targeting young people are seeking blogger collaborations on these emerging platforms. However, current personal media research still tends to focus on certain platforms and lacks research on some emerging platforms with influence [31]. We believe that as an increasing number of platforms emerge, the perspectives on personal media research and marketing strategies should not be limited to specific platforms.

These gaps have driven the development of this study. The purpose of this work is to explore the factors that influence the willingness of fans to continue following a blogger, especially focusing on how to attract more potential fans and generate word-of-mouth effects by strengthening the relationship between the blogger and their followers in order to bring greater long-term value. In the next section, we will introduce relationship marketing theory to strengthen this argument. At the same time, we will focus not only on one personal media platform as our survey target but also on multiple platforms to increase the universality of our research results.

### 2.2. Relationship Mediator Meta-Analytic Framework

To establish our research model, this paper uses the relationship marketing theory model as a theoretical framework for shaping the relationship between media bloggers and their fans. According to the early definition of relationship marketing, the concept of relationship marketing centers around the development of long-term, stable, and mutually satisfying relationships with customers [32]. Grönroos supplemented the concept of relationship marketing by pointing out that direct contact between customers and other stakeholders, as well as customer information management and service systems, are important elements of this strategic level [33]. Berry further emphasized that the focus of relationship marketing is on attracting customers first, which is a prerequisite for building a relationship between the two parties [34]. The early research on relationship marketing focused on exploring the prerequisites for relationship marketing, including enhancing interaction and building attractiveness.

**Figure 1:**
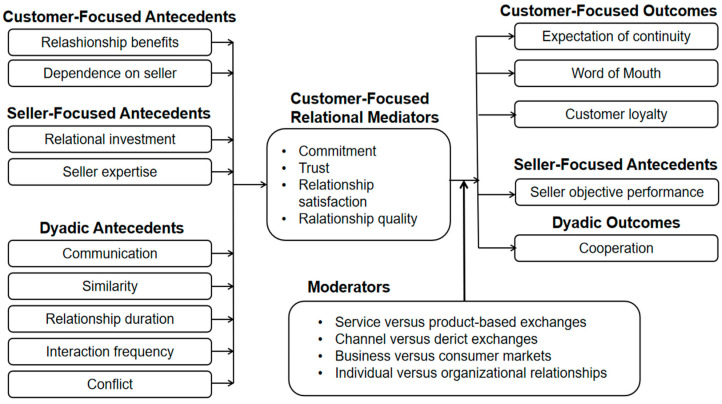
Relational mediator meta-analytic framework.

Palmatier et al. synthesized the previous research on relationship marketing and proposed a relationship mediation model (Figure 1) centered on establishing trust and commitment [22]. The importance of this model lies in its focus not only on the prerequisites for building high-quality relationships, such as communication, dependence, and expertise, but also on key indicators that measure the effectiveness of relationship marketing, such as word of mouth, customer loyalty, and continuity. This makes the framework widely applicable.

Although these studies have revealed the importance of relationship marketing, and the concepts in the relationship intermediary model have been further refined [35], developments in communication technology have led to some changes in the scenario of relationship marketing. For example, contact with customers may be carried out through more efficient means such as personal media. In the context of personal media, the traditional relationship between corporate organizations and customers has transformed into a relationship between personal bloggers and fans. Therefore, the validation of this theory’s applicability in personal media research has driven this study. The relationship mediator meta-analytic framework and important findings in the relationship marketing literature also form the theoretical basis of our study.

In our research, we integrated several important factors from the relationship marketing framework. First, the framework developed for relationship marketing includes several components, including communication, which pertains to the quality, quantity, and frequency of information exchanged between customers and salespeople. Through this theoretical model, we examined the importance of communication mechanisms as a prerequisite for establishing relationships between bloggers and fans in the context of personal media. When bloggers reply to comments from fans and engage in a series of interactive behaviors such as information exchange, they can win over the goodwill of their fans. Fans’ behaviors such as liking, subscribing, and actively commenting on a blogger’s content undoubtedly enhance the blogger’s influence. Secondly, the relationship mediator framework explores the role of the salesperson’s expertise. The expertise of the salesperson is another crucial prerequisite factor, which encompasses their competence, proficiency, and experience. Expertise also applies to the context of personal media, where personal media bloggers who demonstrate their superior knowledge or experience in certain fields are more likely to gain their fans’ trust [36]. Trust, undoubtedly, is the core of the relationship intermediary model and is the key to improving the quality of the relationship.

Secondly, we consider the core concepts of relationship satisfaction and relationship quality in the framework of relationship marketing as social presence, as they represent a positive and subjective feeling [37] that arises when fans are moved by a creator’s content, leading to a sense of satisfaction with the relationship and increased closeness between both parties. It measures the degree of the relationship in the context of personal media. Furthermore, commitment, as a type of belief [32], aligns with the phenomenon of fanship. In the relationship marketing framework, commitment is regarded as the highest level of a relationship, and the pursuit of it by fans toward creators is an indication of the relationship reaching an advanced stage. Therefore, this paper considers social presence and fanship as mediating variables since they influence the establishment of relationships between creators and users [38]. Finally, while the marketing outcomes of word of mouth and loyalty have been fully explored in the existing literature [16,39], some of these studies are still focused on product services or purchase behavior in the context of personal media. As a prerequisite for shaping influence, research on increasing the number of a creator’s fans and their adherence is scarce. This study places word of mouth in the context of online communication and measures customer loyalty through fans’ sustained attention toward creators, which is more in line with the research context of personal media.

### 2.3. Communication

The process of communication is a dynamic exchange of ideas, thoughts, and emotions among individuals within a given environment. This exchange is designed to transmit information and receive feedback in a mutually beneficial and responsive manner. Availability and publicness are important characteristics of online communication [40]. In the realm of personal media, bloggers strive to provide their followers with a diverse range of communication experiences, including features such as comments, private messages, and real-time chats on their platforms. The most significant characteristic of new media, such as online forums, social networks, and blogs, is their ability to facilitate interactive communication between communicators and audiences. This interaction is a crucial aspect in the development of personal media communication and highlights the importance of a two-way exchange in the communication process.

The interaction and communication between communicators and audiences can strengthen the connection between users, making the communication between the information provider and the receivers closer and more stable, further enhancing the sense of social presence. Real-time interaction between fans and bloggers, as well as personalized feedback from bloggers to fans [29], increases the fans’ attention and discussion of the bloggers, further boosting the popularity of the bloggers in virtual communities.

### 2.4. Attractiveness and Expertise

According to Ohanian [18], source credibility is a critical component of source characteristics and comprises three dimensions: attractiveness, reliability, and expertise. The attractiveness of the information source has been shown to have a significant impact on communication effectiveness. An attractive information source evokes positive feelings among consumers and increases their acceptance of the communicated content, resulting in a more favorable brand attitude and greater purchase intentions. Previous studies have also demonstrated the impact of information source characteristics, such as attractiveness and expertise, on consumer purchase intentions and customer engagement [41,42].

When users follow a blogger, they may consider the blogger’s knowledge, experience, and ability. The professionalism of a personal media blogger can provide users with more comprehensive, detailed, and professional information, which can make users feel a sense of identity with the quality and usefulness of the information and reduce their doubts. Users will feel that the blogger is real, reliable, and friendly, rather than just someone who conveys information, thus developing a trusting attitude and becoming loyal users. Therefore, the blogger’s expertise makes fans believe that the content he or she publishes is based on a professional perspective, which shapes the audience’s social subjective experience [8], thus changing the fans’ social presence.

When a communicator is attractive, their audience may be more inclined to accept the information conveyed by the communicator [16]. In the context of personal media, if a blogger has a certain level of attractiveness in appearance, it will first affect the initial impression of fans towards the blogger. Visual aesthetics bring pleasant emotions to fans and give the audience a warm, friendly, and positive feeling. This encourages fans to continue to follow the blogger’s content to satisfy their own needs, which brings them closer together mentally. Although fans may not see the blogger’s appearance as the main driving force for following them, the enhancement of social presence is possible.

In the context of personal media, the attractive force of bloggers is further subdivided into two dimensions: expertise and attractiveness. When a personal media blogger has a strong appeal, it enhances user interest and attention, and stimulates their curiosity and desire to explore, making them attached to the blogger, even leading to fanatical worship and adoration.

### 2.5. Social Presence and Fanship

In 1976, Williams, Short, and Christie introduced the notion of “social presence”, which refers to the degree of perceived interaction and communication with actual individuals through online media [43]. Later, Rice defined social presence as the level of connection established between followers through mediated communication [38]. His study emphasized the profound impact of social presence on the formation of relationships between content creators and their followers. Social presence entails more than merely perceiving the presence of others; it involves establishing a bond between communicators through mutual understanding and intimacy. Online interactions between communicators and audiences can enhance the sense of community and strengthen the relationships between them, which further enhances their social presence. Researchers have explored and confirmed the impact of social presence on network users’ cognition, attitudes, and behavioral intentions in various online activities [36]. Personal media not only allows users to publish information and interact with others through text, pictures, and other forms but also supports information exchange in the forms of voice and video, which is highly beneficial for users who perceive the existence of interactive objects and experience the social presence of “being with others”. The disappearance of physical distance and the illusion of face-to-face communication with personal media influencers can help shorten the psychological distance between users and influencers. When fans feel closer to influencers, they may have a higher level of satisfaction, become more active in sharing influencer content, and pay more frequent attention to their updates.

Fanship is a compelling phenomenon that encompasses the wholehearted involvement and enthusiastic expression of fans in sports, entertainment, fashion, and various other domains of consumption [44]. This fervent sense of dedication not only galvanizes fans but also infuses their lives with passion and joy [45]. Personal media bloggers can satisfy users’ needs and interests by continuously improving their expertise and attractiveness, attracting more attention and participation from users. Users can communicate with other fans through virtual communities, which further enhances a blogger’s influence. Because fans have similar points of identification with the blogger, this also benefits their opinion exchange and emotional attachment and sense of belonging [38]. These benefits may enhance the fans’ willingness to continue following the blogger. In the context of this article, fanship is defined as the extent of followers’ devotion, ardor, and concern towards a personal media blogger, as well as their persistent engagement with the content that the blogger disseminates.

### 2.6. Continued Usage Intention and Word of Mouth

Continued usage intention is a commonly employed construct in studies concerning technology acceptance models or expectation confirmation models (ECM) [46]. In the present study, continued usage intention is defined as a user’s eagerness to persist in following the content disseminated by a personal media blogger and in recommending it to others after experiencing content satisfaction. Word-of-mouth (WOM) communication, which pertains to the face-to-face communication of information or opinions about a product or service, is deemed a critical facet of continued intention to use [47]. Parthasarathy and colleagues pointed out in their research on online services that the cost of acquiring a new user is five times that of maintaining an existing user. This is because the cost of developing five new customers is much higher than maintaining one old customer, and word-of-mouth communication from old customers plays a very important role in improving a company’s performance [48].

Several scholars have also explored the relationship between electronic word of mouth and continued intention to use within the context of the internet [49]. Additionally, a study suggests that subjective perceptions affect users’ attitudes toward data assessment use, resulting in their intentions to use [50]. Consequently, the continued use intention of personal media followers is examined in terms of two dimensions: use intention and word of mouth.

## 3. Research Model and Research Hypothesis

This article draws on the relationship marketing framework proposed by Palmatier et al. (2006) as its theoretical basis [22] and delves into the impact of personal media bloggers as external stimuli on the establishment of relationships between bloggers and users, as well as the impact of the relationships established between personal media bloggers and users on the users’ willingness to continue using the platforms. Building on the attractiveness and expertise of personal media bloggers, as well as the characteristics of the communication between bloggers and users, this article explores the mechanisms by which social presence and fanship act as mediators in the relationships between personal media platforms and users’ continued usage intention.

Furthermore, this article defines social presence and relationship quality in relationship marketing as social immediacy, and it defines fanship as commitment. As such, this article proposes a model that examines the mediating effects of social immediacy and commitment on the relationships between personal media platforms and users’ continued usage intention. Additionally, users’ continued usage intention is measured through both the users’ willingness to continue using the platforms and word-of-mouth communication.

### 3.1. Research Hypothesis

#### 3.1.1. The Effect of Personal Media Blogger Attributes on Social Presence and Fanship

The hypotheses in this section are grounded in information source theory, which posits that the characteristics of information disseminators are critical in shaping consumer attitudes [51]. This theory suggests that the appearance of a disseminator, in terms of attractiveness and credibility, has a significant impact on consumer behavior. Attractiveness emanating from an information source elicits feelings of pleasure, resonance, and aspiration in the audience. Attractiveness refers to the attractive effect that an information source with an outstanding appearance, graceful physique, and tasteful attire has on the audience. Most of the audience enjoys the feeling of beauty and consequently transfers their emotions to the information and products that the information source intends to convey. The more attractive the disseminator, the more reliable and credible the information that they provide is perceived to be, which increases the likelihood that the audience will internalize and accept the information [52]. Therefore, it can be inferred that the attractiveness of personal media bloggers can influence the establishment of relationships between these bloggers and users. The credibility of an information source is determined by its level of expertise and authenticity, with more professional and authentic sources being more likely to have their messages accepted by their audience, according to Kamis [53].

Social presence, as a subjective feeling, describes the extent to which the audience perceives the real existence of the media role psychologically. In media research, social presence, an important factor, is often used to evaluate the ability to convey psychological relevance and warmth through media [54]. When personal media bloggers exhibit their expertise and charm, followers are attracted to these personal characteristics and deeply respond to the trust and affinity of the blogger.

Fanship is a psychological attribute that can explain consumers’ enthusiastic obsessions with a specific object well. It is independent of others’ evaluations and depends on an individual’s subjective judgment to form a concept. Fanship is a dynamic psychological attachment process, which means that this relationship will change over time [55]. This also means that the more the blogger displays the traits that their followers love, the stronger their relationship with their followers will become. Based on these findings, the following hypotheses are proposed:

**H1:**  *Personal media blogger attributes have a significant positive effect on social presence.*

**H1-1:**  *Expertise has a significant positive effect on social presence*.

**H1-2:**  *Attractiveness has a significant positive effect on social presence*.

**H3:**  *The attributes of personal media bloggers have a significant positive effect on fanship.*

**H3-1:**  *Expertise has a significant positive effect on fanship.*

**H3-2:**  *Attractiveness has a significant positive effect on fanship.*

#### 3.1.2. The Effect of Communication on Social Presence and Fanship

Compared with traditional media, the audience of personal media is more convenient and smoother in terms of communication and interaction with the media or with each other. Personal media provide users with a diverse range of experiences, such as sharing, exploring, communicating, and interacting on their own platforms, which satisfies the core purpose of using personal media for the audience. This distinctive characteristic of personal media platforms permits direct communication between followers and bloggers, fostering a more intimate relationship between the two parties. The exchange of information, such as through inquiries, direct messaging, or leaving comments, heightens the interaction between users and bloggers. Therefore, personal media facilitates connections [56], and this direct communication is more likely to create a sense of closeness between followers and the blogger. Through continuous communication with their followers, personal media bloggers can fully understand their preferences and needs and provide valuable products or services, thus gaining the trust of and resonance with their followers, further enhancing the emotional connection between them. If a blogger only presents their own content unilaterally, ignoring interaction with their followers, the impression of the blogger in the fans’ minds will weaken. Therefore, communication is an important prerequisite for bloggers to establish relationships with their fans.

As the occurrence and degree of social presence can be influenced by the ability to transmit information, communication, which enhances information exchange, gives fans a more authentic experience. This not only brings more attention to the fans but also increases user retention. Previous research on Instagram has shown that compared with traditional celebrities, consumers have a stronger sense of social presence for celebrities on Instagram [57]. Based on these findings, the following research hypotheses are postulated:

**H2:**  *Communication has a significant positive effect on social presence.*

**H4:**  *Communication exerts a significant positive influence on fanship.*

#### 3.1.3. The Effect of Social Presence on Word of Mouth and Usage Intention

Social presence compensates for the perceived distance between media bloggers and their audiences, allowing users to perceive the presence of an interactive object and the feeling of being “together with others” during usage. This authentic experience enhances user attention and positively influences their willingness to continue using the platforms [58]. Previous studies on applications have shown that social presence can optimize the consumer experience, increase positive emotions, and subsequently influence continuous usage intentions [59]. We further believe that social presence is an important mechanism that drives continuous usage intention. On the one hand, the authenticity and warmth of the feeling generated via social presence provide followers with a positive motivation to actively promote the blogger, thus generating a word-of-mouth effect. On the other hand, social presence further enhances fan loyalty and strengthens their willingness to follow the blogger. As a result, research hypotheses H5-1 and H6-1 are proposed.

**H5-1:**  *Social presence has a significant positive effect on word of mouth.*

**H6-1:**  *Social presence has a significant positive effect on usage intention.*

#### 3.1.4. The Effect of Fanship on Word-of-Mouth Spread and Willingness to Continue Using

Fanship can explain well the attributes of a consumer’s mindset when they are passionate about and devoted to a particular object. This devotion is independent of the evaluations of others and relies on the consumer’s own subjective judgment [60]. Previous research on the Super Bowl has shown that when the emotions of avid fans are stirred, such as when their team wins, their feedback on advertising becomes more effective [61]. For fans of media influencers, the content posted by these influencers will evoke emotions in their followers, leading to increased word-of-mouth marketing. Furthermore, the higher the number of followers that an influencer has, the higher their level of recognition and loyalty [62], and the stronger their followers’ desire to follow them. The more dedicated fans a personal media blogger has, the more enthusiastic their followers will be about the blogger’s content, and they will be more proactive in supporting the blogger. This leads to the following research hypotheses.

**H5-2:**  *Fanship has a significant positive effect on word of mouth.*

**H6-2:**  *Fanship has a significant positive effect on usage intention.*

### 3.2. Research Model

**Figure 2:**
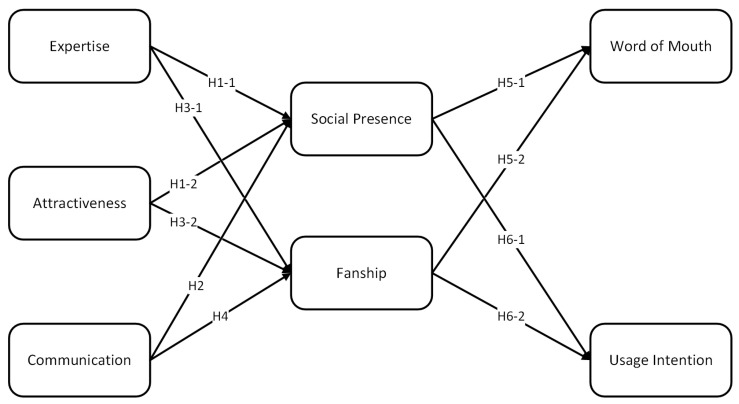
Research model.

In summary, our proposed theoretical model (Figure 2) considers expertise, attractiveness, and communication as the prerequisites for relationship formation. These factors, through the mediating effects of social presence and fanship, influence word of mouth and the continued following intentions of the fan community. As shown in Table 2.

## 4. Research Methodology

### 4.1. Definitions of Variables and Instruments

Based on the aforementioned literature, this study aims to identify the defined dimensions and measures of each variable in the model of personal media bloggers’ influence on their followers’ sustained following. The variables considered include expertise, attractiveness, communication, social presence, and fanship. The measurement items in the questionnaire were adopted by modifying relevant prior research. All constructs were assessed on a 5-point Likert-type scale, ranging from 1 (strongly disagree) to 5 (strongly agree). The source of measurement items in the research model and the definitions of each variable are shown below.

- Expertise (E): Expertise is defined as the professional knowledge and skills of a blogger and is measured using a scale of four items. The measurement dimensions of expertise were adapted from Gilmore and Pine’s study (2007) [63] and were appropriately modified and adjusted based on the characteristics of personal media bloggers, resulting in a final set of 7 items.
- Attractiveness (A): Attractiveness is defined as the beauty and appeal of a blogger’s image and appearance and is measured using a scale of two items. The dimension of attractiveness was measured by adapting and adjusting the measurement items from the study by Kim and Jun (2016) [64], and we included 2 items that were specific to personal media bloggers.
- Communication (C): Communication is defined as the level of interaction and emotional connection between a blogger and their fans and is adapted from two items from the study by Cho and Lim [65].
- Social presence (SP): Social presence is defined as the feeling of closeness between a blogger and their fans when using their personal media platform and is measured with four items from the study by Cho and Lim [65].
- Fanship (F): Fanship is defined as the degree of fanaticism of followers toward bloggers and is measured using a scale of seven items proposed by Lee [66].
- Usage intention (UI): Usage intention is defined as the satisfaction experienced by personal media followers after following bloggers and their desire to continue following them. The measurement dimension of usage intention is based on the questionnaire developed by Kim et al. [67] and was modified to include two items.
- Word of mouth (WOM): Word of mouth is defined as the tendency of personal media followers to recommend the bloggers that they follow to others, thereby generating word-of-mouth effects. The measurement dimension of word of mouth is based on the questionnaire developed by Zeithaml et al. [68] and was adjusted to consist of two items.

### 4.2. Samples and Data Collection

The sampling subjects in this study focused on followers who had utilized personal media and made purchases through the recommendations of personal media bloggers via links, thereby enhancing the reliability of the questionnaire results. The target group consisted of individuals who had practical experience with personal media platforms and currently followed personal media bloggers, which provided valuable data for this research.

To ensure the validity and reliability of the research results, a rigorous questionnaire design process was adopted in this work. The questionnaire was designed based on a combination of theoretical foundations, empirical literature, and logical reasoning, and underwent a pre-testing phase. A convenience sample of 50 personal media followers was selected for a preliminary study, and the collected data were analyzed for reliability and validity. The results show that the scale indicators were consistent with the requirements of this research. The formal questionnaire survey was conducted online, with a survey period from 20 April to 22 April 2023. The data for our research were collected through this online questionnaire. A total of 170 questionnaires were collected, and 14 samples from respondents who did not follow any bloggers were removed, as well as 1 incomplete and invalid questionnaire. This resulted in a total of 155 valid questionnaires, with an effective response rate of 91.18%. This work took the personal media audience currently showing attention to bloggers as the research object, ensuring that the collected data had practical value.

### 4.3. Methodology

In this study, to analyze the validity of the collected data, a combination of the SPSS24.0 and AMOS24.0 software was utilized. A frequency distribution analysis was conducted to gain insights into the demographic characteristics of the respondents. The reliability and validity of the measurement terms, such as the attributes, communication, social presence, fanship, and usage intention of personal media bloggers, were analyzed to verify their effectiveness in expressing the conceptual information and internal consistency of the dimensions. Correlation analysis and structural equation modeling, using the maximum likelihood estimation method, were carried out to validate the structural model of this study. To assess the mediating roles of social presence and fanship, mediation effect analysis based on the bootstrap method was employed.

### 4.4. Research Results

#### 4.4.1. Descriptive Statistics

Demographic characteristics were analyzed using SPSS24.0 based on the basic personal information of 155 respondents (Table 3). The data collected in this study covered 73 male (47.10%) and 82 female (52.90%) participants. The ages were mostly between 20 and 29 years old, accounting for 80% (124 individuals) of the participants. In terms of education, the percentage of respondents with a bachelor’s degree was the highest, at 74.84% (116). Therefore, the gender and age distribution of the survey participants in this study were in line with the research requirements.

In this study, we examined the frequency of personal media use and the average duration per session. The results show that the majority of the participants utilized personal media 7 times or more per week, accounting for 76.77% (119) of the total participants. Meanwhile, only a small proportion of respondents, representing 12.26% (19), used personal media infrequently, less than 2 times per week. In terms of the average duration per session, 41.94% (65) of the participants reported spending from 30 min to less than 2 h on personal media, while 32.26% (50) of the participants used personal media for over 3 h per session. These findings suggest that personal media is becoming increasingly integrated into the daily lives of the population and exerting a growing influence.

#### 4.4.2. Reliability and Validity Analysis

##### CFA of Latent Variables

In order to examine and confirm the convergent validity and discriminant validity of each variable, we used AMOS24.0 to perform a confirmatory factor analysis of the data. After repeated testing, 2 measurement items, “I am willing to pay to follow the information of the bloggers I follow” and “I am willing to provide creative ideas or materials related to the content for the bloggers I follow”, had factor loadings of less than 0.5 and were therefore deleted. The remaining 26 items were reconstructed to validate the measurement model of expertise, attractiveness, social presence, fanship, word of mouth, communication, and usage intention using confirmatory factor analysis in AMOS 24.0. Based on the data presented in Table 3, all model fit indices met the required standards, and the factor loading coefficients were greater than 0.50, indicating good, positive correlations between the observed and latent variables. The X^2^ value was 283.275 (df = 239), the RMSEA was close to 0 at 0.035, the CFI was 0.985 (>0.9), the TLI was 0.979 (>0.9), and the SRMR was 0.059 (<0.08). All judgment criteria were met, indicating the good fit of the measurement model. Additionally, the model’s construct reliability was greater than 0.60, and the AVE was greater than 0.5. The model showed good convergent validity. As shown in Table 4 specifically.

##### Discriminant Validity of Latent Variables

In this paper, the discriminant validity of the latent variables was tested, and the results are shown in Table 5. The square roots of the AVE values for the attractiveness, expertise, communication, social presence, fanship, usage intention, and word-of-mouth variables were all greater than the correlation coefficients between each latent variable, indicating that the discriminant validity of each measure in this paper was good, which provided a basis for the next structural equation analysis.

### 4.5. Hypothesis Testing

**Figure 3:**
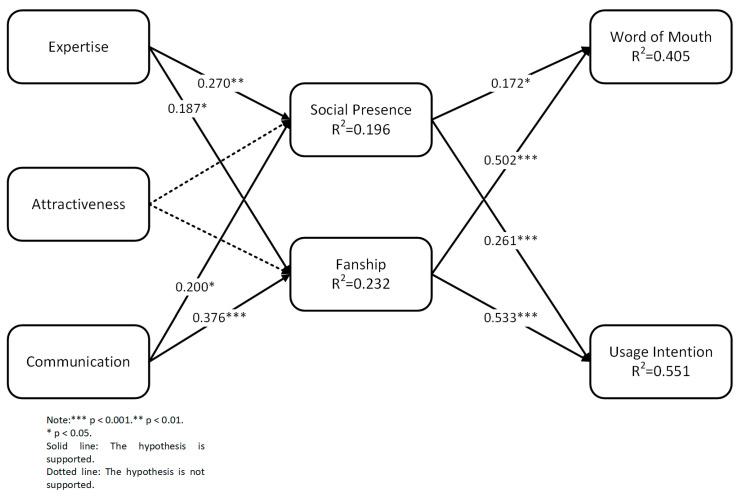
The model of the factors influencing the attributes of personal media bloggers on followers’ continued usage intentions.

The research hypothesis proposed in this work was verified by combining the results of the validation factor analysis to construct a model of factors influencing the followers’ continued usage intention. As shown in Table 6, X^2^ = 6.709 (df = 7), X^2^/df = 0.958 (<3), the RMSEA (0.000) is close to 0, the CFI is 1.000, the TLI is 1.000, and SRMR = 0.024 (<0.05). Thus, all judgment values are greater than 0.9, and the model has a good fit. From the data in Table 6, it can be seen that hypotheses H1-2 and H3-2 are not valid, while the rest of the hypotheses are all valid. As can be seen in Figure 3, there is a significant positive effect of expertise and communication on social presence, and expertise (β = 0.270; *p* < 0.01) has a greater effect on social presence than communication (β = 0.200; *p* < 0.05). There is a significant positive effect of expertise and communication on fanship, and communication (β = 0.376, *p* < 0.001) has a greater effect on fanship than expertise (β = 0.187, *p* ɤ *0*.05). Social presence and fanship have significant positive effects on word of mouth, with fanship (β = 0.502; *p* < 0.001) having a greater impact on word of mouth than social presence (β = 0.172, *p* < 0.05). Social presence and fanship have significant positive effects on usage intention, with fanship (β = 0.533; *p* < 0.001) having a greater impact on usage intention than social presence (β = 0.261; *p* < 0.001). Social presence and fanship can jointly explain 40.5% of the variation in word of mouth. Social presence and fanship can jointly explain 55.1% of the variation in usage intention.

### 4.6. Mediation Effect Test

By examining the mediating effects of social presence and fanship, we can reveal the mechanism of the influence of personal media bloggers on their followers. In this article, we use the bias-corrected bootstrap (1000 times) method to test the mediating effects of social presence and fanship between expertise, communication, and usage intention. The mediating effect holds if the confidence interval does not contain 0. Expertise → social presence → usage intention (β = 0.071); Communication → social presence → usage intention (β = 0.052); expertise → social presence → word of mouth (β = 0.047); communication → social presence → usage intention (β = 0.034); and social presence fully mediates the effects of expertise and communication on usage intention and word of mouth. Expertise → fanship→ usage intention (β = 0.100); communication → fanship → usage intention (β = 0.200); expertise → fanship→ word of mouth (β = 0.094); communication → fanship→ word of mouth (β = 0.189); and fanship fully mediates the effects of expertise and communication on usage intention and word of mouth. The specific content is shown in Table 7.

## 5. Discussion

### 5.1. Key Findings

This study aimed to investigate the factors influencing followers’ continuous use of personal media. A model based on relationship marketing theory was constructed and tested to reveal the behavioral mechanisms of influencer characteristics that affect followers’ continued following, interest, and loyalty. The results show that followers’ continuous following behavior is influenced by social presence and fanship, and influencers’ expertise significantly influences these factors, with the influence of expertise being particularly significant. This finding supports the results of social media marketing research [16]. The impact of attractiveness on social presence and fanship is not significantly positive. Emotions serve as a link in interpersonal relationships, and even if personal media bloggers possess charm, they may fail to grasp the users’ emotions, resulting in a lack of intimacy.

The findings also reveal that communication plays a crucial role in establishing relationships in the era of the internet. Personal media users have the opportunity to not only view the content presented by bloggers but also interact with them by liking and commenting on it. The timelier the response from a personal media blogger, the stronger the user’s desire to interact, and the easier it is for the personal media blogger to establish a close connection with the user. Therefore, it is important for personal media bloggers to maintain constant interaction and communication with their users. Each positive emotional communication enhances user satisfaction, strengthens their trust in the personal media blogger, and results in an increased number of fans.

The survey sample of this study mainly focused on young people aged 20–29. In the initial survey, we found that most young people use various types of social media. Therefore, we expanded the scope beyond a specific personal media platform to make our conclusions more universal. In addition, most of the sample used personal media more than once a day, which is obviously advantageous for accurately tracking the establishment and sustainability of relationships and researching the intention to continue using personal media.

The significant influence of expertise indicates that followers are more easily impressed by high-quality content in a blogger’s professional field, which promotes the formation of a social presence and a fan atmosphere among followers. This effectively influences their following and word-of-mouth willingness. The formation of a fanship culture has a strong influence on willingness to follow. The results show that for bloggers who pursue a stable fan base, maintaining this atmosphere can effectively reduce follower loss. In addition, the mediation analysis shows that social presence and fan relationships mediate the influences of expertise and communication on the willingness to continue using, which further supports the important role that these two variables play in the formation and maintenance of relationships.

### 5.2. Theoretical Contributions

This paper contributes to the research on the impact of continued usage in the personal media marketing literature, utilizing Palmatier’s relationship marketing framework. This study offers a valuable perspective to enable bloggers to establish and strengthen their connections with their followers. The findings demonstrate that expertise and communication play crucial roles in building relationships between bloggers and followers and that the relationships between personal media bloggers and their followers affect the followers’ intentions to continue following them. Therefore, the relationship marketing framework can serve as a significant guide for the development of personal media operations and provide a theoretical basis for future research in this area. Furthermore, this study showcases the versatility of relationship marketing as it can be applied to multiple scenarios [69].

This study also provides empirical validation of the significance of fanship in the personal media marketing process. While previous studies have employed this concept as an indicator to scrutinize team identification [70,71], they have not examined the role that fanship plays in depth in the personal media field. Furthermore, a comparative analysis of fanship as a mediating variable revealed that it has a more substantial impact on word of mouth and usage intention than social presence. This implies that fanship is a variable possessing robust predictive power and can serve as an essential indicator for the forecasting of consumer behavior.

This study also extends the application of the information source model to the personal media environment. The expertise identified from the model revealed a strong connection between bloggers and followers, highlighting that the demonstration of a blogger’s exceptional expertise can be highly effective in fostering the establishment and progression of their relationship with their followers. This is because attributes function as antecedents that enhance fanship and, consequently, influence personal media usage intention. Thus, the results of the information source model can be employed in studies aimed at enhancing the relationships between influencers and their followers.

This research also offers a valuable contribution to the study of the metaverse, as an increasing number of scholars are integrating metaverse techniques into the examination of marketing strategies. For instance, some researchers use the attributes of avatars in created digital space settings to achieve enhanced word-of-mouth and usage intention effects [72]. These findings aid scholars in comprehending and constructing empirical sources of brand communication and consumer behavior in an era of engagement, immersion, and feedback value creation.

### 5.3. Implications for Practice

The primary practical significance of this study lies in providing marketers with a set of criteria for the selection of suitable influencers to endorse their brands or products. Although previous research on personal media platforms has helped marketers to identify influential factors on a macro level [73,74], this research provides a perspective based on relational mechanisms. Specifically, expertise and communication have significant positive impacts on social presence and fan engagement. This implies that the more skilled and knowledgeable a blogger is, the more likely they are to gain users’ favor and develop positive relationships. Therefore, if brands choose to collaborate with bloggers, they should provide them with adequate product training. Personal media bloggers have the ability to create compelling content that resonates with users, evokes emotions, and fosters user loyalty. Therefore, the quality of the content is essential to increase a blogger’s following. Personal media bloggers should dedicate themselves to creating high-quality content consistently in order to build a loyal following.

The study results indicate that bloggers’ expertise indirectly affects word of mouth through the mediation of social presence and fanship. While social presence and fanship have a partial mediating effect, they fully mediate the effect of expertise on usage intention. These results are in line with the conclusions drawn by Ohanian [18], who stated that the credibility of endorsers depends on their attractiveness, reliability, and expertise, with expertise being the most important factor affecting purchase intentions. The impact of fanship on both word of mouth and usage intention is more significant than that of social presence. The connections forged between bloggers and users have a profound effect on the users’ willingness to continue using the platforms and their ability to generate valuable word of mouth. The intimacy of these connections enhances users’ commitment to bloggers and leads to more effective word of mouth. Thus, a strong relationship between a blogger and their followers is a crucial factor in achieving success. As such, blog authors should focus on cultivating fan relationships and continuously improving their professional skills and content quality to stand out and elicit positive behavioral responses from their audience. Based on the findings of this study, the following practical implications can be drawn for various stakeholders.

Personal media bloggers should focus on building their expertise in their respective fields and strive to consistently provide high-quality content to their followers. This will help them to establish a strong social presence and foster a fanship culture among their followers. Personal media bloggers should maintain regular communication and interaction with their followers. Prompt responses to comments and messages can help to build intimacy and trust, resulting in an increased number of fans and a lower follower loss rate.

Marketers who work with personal media bloggers should prioritize expertise over attractiveness when selecting influencers for marketing campaigns. By leveraging the expertise of influencers, marketers can ensure that the content that they promote is of high quality and relevant to their target audience. Marketers should also encourage personal media bloggers to engage in fan communities and interactions with their followers to help establish long-term relationships.

Personal media platforms can improve their users’ interaction behaviors by providing more opportunities for interaction and communication between bloggers and followers, such as adding features that enable live streaming, Q and A sessions, and polls.

### 5.4. Limitations and Future Research Suggestions

This study has several limitations that must be acknowledged. Firstly, the study identifies several factors that affect the relationships between personal media bloggers and their followers, but the external variables in this study are limited to the characteristics of the bloggers. To expand the research topic based on this study, a broader investigation or comparative study could be conducted. For instance, when expanding the research topic, the study could explore factors that affect relationship building beyond the characteristics of advertising personal media bloggers and include variables related to users, media, and other factors. This study does not consider followers’ motivations for following bloggers. Such motivations can be divided into entertainment, social, and functional categories, and they can impact followers’ expectations and preferences for video content and personal media bloggers. Additionally, besides attractiveness and expertise, empathy may also play a role in followers’ intentions to continue following a blogger. It would be useful to conduct a specific comparative study on users of different personal media platforms, such as Facebook, YouTube, and TikTok.

Secondly, due to the limited prior research on communication and fanship variables, although the variable design of the questionnaire was based on some references, it was still not comprehensive enough. Therefore, in future research, a more in-depth and professional study can be conducted on the design of communication variables, providing more reliable support for subsequent quantitative research related to communication.

Thirdly, this article mainly collected data by distributing questionnaires on online platforms. According to the data sample results, there were two deficiencies in the questionnaire survey. Firstly, the source of the questionnaire was mainly within China. Therefore, in future research, the geographical scope of data collection can be expanded to enhance the diversity and decentralization of sample data sources. Secondly, the majority of the surveyed subjects were aged 20–29, accounting for 80% (124 people), followed by those under 20, accounting for 37.42% (28 people). Although this has a certain representativeness, as the elderly personal media user group increases, the sample age range can be expanded in future research to further improve the universality of the research conclusions.

Finally, with the rapid development of metaverse technology, future research should continue to investigate how metaverse technology can be leveraged to create engaging, interactive, and memorable brand experiences for consumers. Additionally, scholars should also focus on understanding how different types of metaverse environments and virtual characters can influence consumer behavior and how companies can leverage these insights to develop targeted marketing strategies that resonate with their target audiences.

## Figures and Tables

**Table 1:** Summary of the key literature on personal media influencers.

Source	Focus of Content	Method	Main Variables	Key Findings
Stubb et al. (2019) [25]	To investigate the effects of stressing objectivity in influencer product posts on consumer reactions.	Experiment	Landing page, disclosure type, and purchase intention	When participants are exposed to an impartiality declaration and then sent to a product page rather than a start page, their brand attitudes and purchase intentions are less likely to change. Whether or not consumers view the information as advertising will determine whether or not an impartiality disclosure is successful.
Kay et al. (2020) [26]	The effect of small- and large-scale influencers, as well as the disclosure of sponsored native advertising, on consumer opinions of goods.	Experimental method with a 2 × 2 factorial design	Micro-influencers, macro-influencers, sponsorship disclosure, and purchase behavior	In comparison with macro-influencers (those with more followers), micro-influencers (those with fewer followers) are more effective at endorsing goods. Disclosure of sponsored content can improve results for brands.
Zhou et al. (2021) [28]	To investigate the value of social media influencers’ (SMIs) narrative techniques in resolving potential influencer marketing problems such cultural differences, conflicts between work and personal life, and unfavorable views of sponsorship disclosure.	Focus group interviews	-	The narrative strategies used by social media influencers (SMIs), such as brand attribute analysis, brand love inspiration, and self-identity construction, can be useful in resolving these problems.
Leite et al. (2022) [30]	To investigate the impact of social media influencers’ personal disclosure on their credibility.	Experiment		Excessive intimate self-disclosure by social media influencers may harm their credibility; however, if the disclosure is appropriate, it can boost their credibility by satisfying their followers’ need for connection.
Ooi et al. (2023) [29]	The aim was to examine the elements that influence consumers’ perceptions of social media influencers and the products or services that they promote, and to determine how these perceptions translate into actual purchasing decisions.	Survey	Influencer credibility, interactivity, and purchase behavior	Factors such as mobile convenience, influencer credibility, and interactivity play a significant role in shaping consumers’ attitudes toward social media influencers and advertised products or services, ultimately leading to purchase behavior. The impact of the attitudes toward social media influencers on purchase behavior is fully mediated by the attitude toward the products or services. The relationship between influencer credibility and attitude towards a product or service is significantly moderated by gender.

**Table 2:** Summary of theoretical constructs.

Research Model	Specific Factors	Description	Construct in the Relationship Marketing Framework	Supporting Rationale
Antecedents	Expertise	The blogger demonstrates superior knowledge or experience in a specific field.	Seller expertise	The ability and experience of salespeople have a beneficial effect on attracting customers.
	Attractiveness	The blogger’s physical appearance.	-	
	Communication	The blogger’s interactions with fans through real-time chat, replies, comments, and likes.	Communication	The quantity, quality, and frequency of information transmission influence the formation of valuable relationships.
Mediators	Social Presence	The positive and warm subjective feelings that fans have towards the blogger.	Relationship satisfaction Relationship quality	The key factor in the relational mediator meta-analytic framework for the formation of long-term and stable relationships.
	Fanship	The admiration, worship, and even belief in the blogger by fans.	Commitment	
Performance Outcomes	Word of Mouth	Fans actively recommend and share the blogger’s content with others.	Word of mouth	Customers actively and positively promote potential customers to salespeople.
	Usage Intention	Fans’ willingness to continue following the blogger.	Customer loyalty	Customers’ long-term purchase and use of a company’s products or services promoted by salespeople.

**Table 3:** Demographic characteristics of the study sample.

Variable	Category	Frequency	Percentage
Gender	Male	73	47.10
	Female	82	52.90
Age	Below 20	28	18.06
	20–29	124	80
	30–39	3	1.94
Education	Diploma	2	1.29
	Bachelor’s	116	74.84
	Master’s	37	23.87
Geography	First- and second-tier cities	36	23.22
	Third- and fourth-tier cities	61	39.35
	Small cities below the fourth tier	58	37.41
Weekly frequency of use of personal media	Less than once a week	13	8.39
	1–2 times a week	6	3.87
	3–4 times a week	7	4.52
	5–6 times a week	10	6.45
	7 or more times a week	119	76.77
Average duration of each personal media use	Less than 30 min	25	16.13
	30 min to 1 h	32	20.65
	1–2 h	33	21.29
	2–3 h	15	9.68
	3 h or more	50	32.26

**Table 4:** CFA results of latent variables.

	Statement	Standardized Estimate	C.R.	CR	AVE
Expertise	The bloggers I follow have strong expression ability.	0.875	13.777	0.940	0.693
	The bloggers I follow are articulate speakers.	0.886	13.935		
	The bloggers I follow are likeable.	0.927	15.149		
	The bloggers I follow have clear content.	0.887	14.143		
	The bloggers I follow have professional content.	0.768	11.402		
	The bloggers I follow are trustworthy.	0.751	11.482		
	The bloggers I follow are very real.	0.705	10.229		
Attractiveness	The bloggers I follow have a good sense of style.	0.888	14.112	0.890	0.801
	The bloggers I follow are attractive.	0.902	13.772		
Communication	The bloggers I follow frequently reply to comments (including messages).	0.701	8.942	0.675	0.509
	The bloggers I follow often show their private lives to the audience.	0.727	9.247		
Social Presence	I feel like I’m having face-to-face communication with my blogger.	0.740	8.088	0.814	0.530
	I feel like I am in the same space as the bloggers I follow.	0.500	7.902		
	I feel happy when I think of the bloggers I follow.	0.770	13.376		
	I feel a sense of belonging to the platform where the bloggers I follow are located.	0.812	12.448		
Fanship	I often check to see if the bloggers I follow have updated their content.	0.673	9.160	0.880	0.515
	I am interested in the information from the bloggers I follow.	0.824	12.219		
	Sometimes, I talk to my friends about the bloggers I follow.	0.787	11.617		
	Even if the bloggers I follow switch to another personal media platform, I will still search or follow them on other platforms.	0.569	7.616		
	Sometimes, I want to like or comment on the opinions of the bloggers I follow.	0.723	10.032		
	The content of the bloggers I follow influences me more or less.	0.726	10.184		
	I would recommend the bloggers I follow to my friends.	0.693	9.759		
Word of Mouth	I share with my friends the content shared by the bloggers I follow.	0.904	14.753	0.904	0.825
	I share with my friends the content of the bloggers I follow.	0.912	14.320		
Usage Intention	I am willing to pay to follow the information of the bloggers I follow.	0.799	11.037	0.707	0.549
	I am willing to watch the advertisements that appear in the content shared by the bloggers I follow.	0.677	9.266		
X^2^ = 282.375 (df = 239), CFI = 0.985, RMSEA = 0.035, TLI = 0.979, SRMR = 0.059.					

**Table 5:** Discriminant validity test of latent variables.

Variable	A	E	C	SP	F	UI	WOM
A	0.895						
E	0.411	0.837					
C	0.404	0.600	0.713				
SP	0.234	0.407	0.379	0.728			
F	0.152	0.381	0.457	0.713	0.718		
UI	0.148	0.363	0.442	0.641	0.690	0.741	
WOM	0.117	0.259	0.330	0.530	0.625	0.521	0.908
SD	0.757	0.601	0.716	0.450	0.602	0.579	0.565
M	3.910	4.025	3.603	3.732	3.623	3.527	3.850

Note: A denotes attractiveness, E denotes expertise, C denotes communication, SP denotes social presence, F denotes fanship, UI denotes usage intention, and WOM denotes word of mouth.

**Table 6:** Hypothesis testing.

Hypothesis	Path	Standardized Estimate	S.E.	C.R.	*p*	Hypothesis Supported
H1-1	Expertise → social presence	0.270	0.069	2.912	0.004	Yes
H1-2	Attractiveness → social presence	0.042	0.048	0.517	0.605	No
H2	Communication → social presence	0.200	0.058	2.161	0.031	Yes
H3-1	Expertise → fanship	0.187	0.091	2.067	0.039	Yes
H3-2	Attractiveness → fanship	−0.077	0.063	−0.969	0.332	No
H4	Communication → fanship	0.376	0.076	4.157	***	Yes
H5-1	Social presence → WOM	0.172	0.111	1.946	0.050	Yes
H5-2	Fanship → WOM	0.502	0.083	5.669	***	Yes
H6-1	Social presence → usage intention	0.261	0.099	3.394	***	Yes
H6-2	Fanship → usage intention	0.533	0.074	6.925	***	Yes
X^2^ = 6.709 (df = 7; X^2^/df = 0.958), CFI = 1.000, RMSEA = 0.000, TLI = 1.000, SRMR = 0.024.						

Note: *** *p* < 0.001.

**Table 7:** Tests of mediating effects.

Path	Standardized Estimate (β)	Boot SE	95% Confidence Interval (LLCI, ULCI)	Result
Expertise → social presence → usage intention	0.071 *	0.039	(0.012, 0.172)	Pass
Communication → social presence → usage intention	0.052 *	0.035	(0.007, 0.162)	Pass
Expertise → social presence→ WOM	0.047 *	0.032	(0.001, 0.125)	Pass
Communication → social presence→ WOM	0.034 *	0.025	(0.002, 0.104)	Pass
Expertise → fanship → usage intention	0.100 *	0.042	(0.030, 0.239)	Pass
Communication → fanship→ usage intention	0.200 **	0.059	(0.099, 0.333)	Pass
Expertise → fanship → WOM	0.094 *	0.066	(0.030, 0.222)	Pass
Communication → fanship → WOM	0.189 *	0.063	(0.082, 0.331)	Pass

Note: * *p* < 0.05; ** *p*< 0.01.

## Data Availability

Data will be provided on request.
